# Diagnostic Potential of Plasma Extracellular Vesicle miR-483-3p and Let-7d-3p for Sepsis

**DOI:** 10.3389/fmolb.2022.814240

**Published:** 2022-02-02

**Authors:** Guanguan Qiu, Jiajie Fan, Guoping Zheng, Jiangping He, Fangping Lin, Menghua Ge, Lanfang Huang, Jiangmei Wang, Jie Xia, Ruoqiong Huang, Qiang Shu, Jianguo Xu

**Affiliations:** ^1^ Shaoxing Second Hospital, Shaoxing, China; ^2^ Department of Thoracic and Cardiovascular Surgery, Children’s Hospital of Zhejiang University School of Medicine and National Clinical Research Center for Child Health, Hangzhou, China

**Keywords:** miRNA–microRNA, miR-483-3p, let-7d-3p, sepsis–diagnostics, extracellular vehicles (EVs)

## Abstract

**Background:** microRNAs (miRNAs) from circulating extracellular vesicles (EVs) have been reported as disease biomarkers. This study aimed to identify the diagnostic value of plasma EV-miRNAs in sepsis.

**Methods:** EVs were separated from the plasma of sepsis patients at admission and healthy controls. The expression of EV-miRNAs was evaluated by microarray and qRT-PCR.

**Results:** A preliminary miRNA microarray of plasma EVs from a discovery cohort of 3 sepsis patients at admission and three healthy controls identified 11 miRNAs with over 2-fold upregulation in sepsis group. Based on this finding, EV samples from a validation cohort of 37 sepsis patients at admission and 25 healthy controls were evaluated for the expression of the 6 miRNAs relating injury and inflammation *via* qRT-PCR. Elevated expression of miR-483-3p and let-7d-3p was validated in sepsis patients and corroborated in a mouse model of sepsis. miR-483-3p and let-7d-3p levels positively correlated with the disease severity. Additionally, a combination of miR-483-3p and let-7d-3p had diagnostic value for sepsis. Furthermore, bioinformatic analysis and experimental validation showed that miR-483-3p and let-7d-3p target pathways regulating immune response and endothelial function.

**Conclusion:** The present study reveals the potential role of plasma EV-miRNAs in the pathogenesis of sepsis and the utility of combining miR-483-3p and let-7d-3p as biomarkers for early sepsis diagnosis.

## Introduction

Sepsis, which is defined as life-threatening organ dysfunction as the result of dysregulation of host response to an infection, is the primary cause of hospital death in the intensive care units (ICU). The annual global burden of sepsis is estimated to be approximately 50 million cases and more than 10 million deaths (Rudd et al., 2020). During the initial phase of sepsis, systemic activation of monocytes/macrophages and neutrophils results in persistent inflammatory response and release of a variety of proinflammatory cytokines, known as the cytokine storm (Delano and Ward, 2016). The cytokine storm and secondary immunosuppression often culminate in multiple organ failure, the leading cause of mortality (Delano and Ward, 2016). Early diagnosis and management of sepsis are essential to reduce organ failure and death through prompt antibiotics, pressors, and adjuvant treatment (Marik and Farkas, 2018).

Traditional biomarkers for sepsis, such as procalcitonin (PCT), IL-6, and c-reactive protein (CRP) have limited discriminative value due to inadequate specificity and sensitivity (Pierrakos et al., 2020). In recent years, microRNAs (miRNAs) have been reported as biomarkers for a variety of diseases. miRNAs are small non-coding 18–25 nucleotide RNAs, which interfere with the expression of up to 30% of protein-coding genes in mammalian cells (Bartel, 2004). miRNAs complement with sequence of target mRNAs, commonly in the 3′ untranslated region, and block protein expression of the target genes *via* translational inhibition and/or mRNA decay (Bartel, 2018). Circulating miRNAs from serum or plasma have been examined as diagnostic and prognostic biomarkers for sepsis (Giza et al., 2016). For example, serum miR-150 was shown as a predictor for survival for patients with critical illness and sepsis (Roderburg et al., 2013). Elevated levels of serum miR-133a were an independent predictor for mortality in sepsis patients (Tacke et al., 2014). Plasma miR-15a and miR-27a levels were significantly reduced while levels of miR-34a were increased in patients with septic shock. The combined expression of the three miRNAs was a strong predictor for septic shock (Goodwin et al., 2015).

Extracellular vesicles (EVs) are functional vesicles produced by almost all human cells and found in various body fluid. EVs were originally regarded as a disposal mechanism for cells, but are now increasingly accepted as important vehicles for intercellular communication (Yanez-Mo et al., 2015; Zhang et al., 2019). EVs are composed of a lipid bilayer specific for their parental cells and carry cargos such as proteins, mRNA, miRNA, and DNA (Islam et al., 2012). The contents of EV cargoes differ significantly depending on the origin and state of cells. EVs are capable of transferring the functional miRNAs and proteins to target cells and duplicating the biological effects of their parental cells (Qiu et al., 2018). Synergies between EVs and autophagy machinery play an essential role in cellular homeostasis and tumor metastasis (Salimi et al., 2020). Application of mesenchymal stem cell–derived EVs has been tested in ischemic diseases (Babaei and Rezaie, 2021), while EVs from dendritic cells have been examined in cancer immunotherapy (Nikfarjam et al., 2020). Our group reported that EVs from aging and young mesenchymal stem cells had differential effects in acute lung injury (Huang et al., 2019). In addition, mesenchymal stem cell–derived EVs alleviated acute lung injury *via* transfer of miR-27a-3p (Wang et al., 2020).

In recent years, EVs have been investigated as markers for diseases. Tissue factor in plasma EVs was crucial for the activation of the coagulation system and served as a biomarker for venous thromboembolism in cancer patients (Ender et al., 2020). Plasma EV protein levels of polygenic immunoglobulin receptor, cystatin C, and complement C5a were independently associated with acute coronary syndrome (de Hoog et al., 2013). Circulating EV-miRNAs, including miR-21, miR-23a, miR-1246, and miR-92a, were reported as novel diagnostic biomarkers for colorectal cancer (Desmond et al., 2019). miR-451a and miR-21-5p in plasma EVs were significantly reduced in Alzheimer’s disease compared to dementia with Lewy bodies. Receiver operating characteristic (ROC) curve analysis suggested that miR-451a and miR-21-5p were potential biomarkers to discriminate between the two diseases (Gamez-Valero et al., 2019).

In the present study, we evaluated miRNA profiles of plasma EVs from sepsis patients at admission and healthy controls. Microarray and RT-PCR analysis showed that levels of miR-483-3p, let-7d-3p, and miR-92b-5p were upregulated in sepsis patients compared with controls. A combination of miR-483-3p and let-7d-3p provided robust value for sepsis diagnosis. Pathway analysis revealed that miR-483-3p and let-7d-3p in plasma EVs may modulate immune response and endothelial function during the early state of sepsis.

## Materials and Methods

### Study Subjects

The discovery and validation cohorts included 3 and 37 sepsis patients, respectively. All sepsis patients were admitted to the ICU of Shaoxing Second Hospital, between November 2019 and January 2020, were consecutively enrolled into our study. All sepsis patients fulfilled the criteria of Sepsis-3 [infection and an acute increase ≥2 in sequential organ failure assessment (SOFA)-score] (Singer et al., 2016). Eligible patients (≥18 years of age) were diagnosed with sepsis within 24 h of admission. The exclusion criteria included the following: age less than 18, cancer, pregnancy, chronic inflammatory diseases, traumatic brain injury, HBV/HCV/HIV infection, and refusal of consent. The discovery and validation cohorts also included 3 and 25 healthy controls, respectively. The healthy controls were age-matched volunteers with normal body temperature (36.4°C–37.2°C) and no evidence of concurrent infection as assessed by physical examination. Written informed consent was acquired from all enrolled patients and healthy volunteers. The study was preapproved by the ethics committee at Shaoxing Second Hospital and conducted in accordance with the principles outlined in Declaration of Helsinki. Clinical data and demographic information for septic patients were extracted from the electronic medical records. Approximately 20 ml of whole peripheral blood (10 ml of plasma) were obtained within 24 h after ICU admission for sepsis patients or immediately after written informed consent for healthy controls.

### Mouse Model of Cecal Ligation and Puncture (CLP)

C57BL/6 male mice (aged 6–8 weeks) were acquired from Shanghai Laboratory Animal Center (Shanghai, China) for the study. The animal study had received approval from the Institutional Animal Care and Use Committee at Zhejiang University School of Medicine and was conducted in accordance with the animal experimentation guidelines. Mice were subjected to polymicrobial sepsis *via* CLP surgery as previously described (Zheng et al., 2020). Mice were anesthetized with phenobarbital (50 mg/kg) *via* intraperitoneal injection. A ventral midline incision (approximately 2 cm) was performed to allow good exposure of cecum. The cecum was tightly ligated at 1 cm from the cecal tip using 3–0 silk suture, punctured twice with a sharp 22-gauge needle, and gently squeezed to extrude a small amount of fecal material. The cecum was repositioned to the peritoneal cavity and the incision was sutured in two layers. For the sham group, surgery was performed to expose the cecum without ligation and puncture before abdominal closure in the animals. To maintain the hydration of the animals, isotonic saline solution was administered subcutaneously (1 ml/mouse). Three of the eight mice in the CLP group, while none of the five mice in the sham group, died within 24 h after surgery. Mice were sacrificed at 24 h after CLP or sham procedure. Blood samples were collected for further analysis.

### Isolation of EVs From Plasma

Plasma from sepsis patients and healthy controls (10 ml) was diluted 1:2 with cold phosphate-buffered saline (PBS) and followed by centrifugation at 2000 g for 30 min to remove cellular debris. The supernatant was diluted 1:5 with PBS and subjected to further centrifugations: 14,000 g for 30 min at 4°C to remove large membrane vesicles and 120,000 g for 2 h at 4°C using a swinging bucket rotor (Optima XPN- 80 centrifuge, SW 32 Ti rotor, Beckman Coulter). The sediment was washed with PBS and centrifuged again at 120,000 g at 4°C for 2 h. The resulting EV pellet was resuspended in 100 µl of cold PBS for RNA extraction and EV characterization. Protein concentration of the resulting EVs was assayed by Pierce BCA Protein Assay Kit (Thermo Fisher Scientific, Waltham, MA).

### Transmission Electron Microscopy (TEM)

Fresh EV pellets were isolated as mentioned above and diluted with PBS. EV samples were absorbed onto 200 mesh copper grids coated with formvar. The grids were negatively stained by a 2% aqueous uranyl acetate solution for 1 min and dried at room temperature. The grids were imaged on a transmission electron microscope (FEI Tecnai G2 Spirit, Hillsboro, OR) operating at 120 kV to view the EVs.

### Nanoparticle Tracking Analysis

EV samples were diluted into appropriate concentrations with PBS. Diluted samples were injected into the analysis chamber of a ZetaView PMX 110 (Particle Metrix, Meerbush, Germany) for particle size and concentration analysis according to the operating instructions. Duplicates were measured for each sample with the same instrument settings. Results were analyzed using the ZetaView software version 8.2.30.1 with a least size of 10, a largest size of 1,000, and a least brightness of 20.

### Microarray Analysis

Plasma EVs obtained from 3 sepsis patients and healthy controls were preserved with TRIzol (Thermo Fisher Scientific, Watham, MA). Small RNAs were isolated using the mirVana miRNA Isolation Kit (Ambion, Austin, TX) by following the manufacturer’s protocol. Microarray procedures were carried out using the miRCURY LNA Array platform (Exiqon, Vedbaek, Denmark). Data were acquired *via* GenePix Pro 6.0 software (Molecular Devices, San Jose, CA). After normalization, potential differentially expressed miRNAs of the two groups were recognized *via* fold change. The threshold value to define a potential upregulation of miRNAs was a 2-fold increase. Finally, a heat map was generated to present distinguishable miRNA expression profiling among the 6 samples.

### Real-Time Quantitative Reverse Transcriptase Polymerase Chain Reaction (qRT-PCR)

Total RNA was isolated from plasma EVs using the TRIzol Reagent. qRT-PCR primers for miR-483-3p, miR-328-3p, let-7d-3p, miR-92b-5p, miR-381-3p, and miR-210-5p were purchased from Genecopoeia (Rockville, MD). cDNA was generated from miRNAs using Mix-X™ miRNA First Strand Synthesis Kit (Takara Bio, Kusatsu, Japan). PCR step was conducted using a Mir-X miRNA qRT-PCR SYBR Kit (Takara Bio) in a LightCycler 480 II system (Roche, Basel, Switzerland). Levels of all miRNAs were quantitated using the ΔΔCt method and normalized to spike-in cel-miR-39-3p (Ribobio, Guangzhou, China). For mRNA analysis, reverse transcription was conducted *via* the PrimeScript™ RT Reagent Kit (Takara Bio). PCR was performed using SYBR Green™ Premix Ex Taq™ (Takara Bio). All target genes were normalized to β-actin mRNA using the standard ΔΔCt method. The primer sequences used are listed as follows: IGF1 forward 5′GCT​CTT​CAG​TTC​GTG​TGT​GGA3′, IGF1 reverse 5′GCC​TCC​TTA​GAT​CAC​AGC​TCC3′; PRKAR1A forward 5′GCA​GCC​TTC​GAG​AAT​GTG​A3′, PRKAR1A reverse 5′TGC​ACA​ACT​GCA​CAA​TAG​AAT​CT3′; PRKACB forward 5′CAT​GCA​CGG​TTC​TAT​GCA​G3′, PRKACB reverse 5′GTC​TGT​GAC​CTG​GAT​ATA​GCC​TT3′; β-actin forward 5′CGT​TGA​CAT​CCG​TAA​AGA​CC3′, reverse 5′AAC​AGT​CCG​CCT​AGA​AGC​AC3′.

### Western Blot Analysis

Plasma EVs were lysed with an appropriate volume of lysis buffer (10 mM Tris-HCl, pH 7.4, 1 mM EDTA, 150 mM NaCl, 0.5% Nonidet P (NP)-40, 1 mM NaN3, and 1 mM PMSF) at 4°C for 30 min. The protein concentration was assayed using a Pierce BCA protein assay kit (Thermo Fisher Scientific). Protein extracts (20 μg) was resolved on 12% sodium dodecyl sulfate-polyacrylamide gel electrophoresis in Tris-Glycine running buffer and transferred to polyvinylidenefluoride membranes (Millipore, Billerica, MA). The membranes were blocked in a blocking buffer consisting of 5% milk in Tris-buffered saline with Tween-20 for 1 h at room temperature and probed with the primary antibodies to CD63 (ab193349, Abcam, Waltham, MA) or CD81 (sc-166029, Santa Cruz Biotechnology, Dallas, TX) in blocking buffer overnight at 4°C. The membranes were then washed 3 times with wash buffer (Tris-buffered saline with Tween-20) and incubated with horseradish peroxidase-conjugated secondary antibodies in blocking buffer for 1 h at room temperature. Following three washes with wash buffer and one wash with PBS, the signals were detected *via* chemiluminescence with the ECL kit (Thermo Fisher Scientific).

### Bioinformatic Analysis

Prediction of miRNA target genes was performed using miRDB and TargetScan databases. Then, KEGG pathway analysis was conducted to determining the pathways regulated by the target genes (https://www.kegg.jp). Statistically significant pathways were represented by the *p* value <0.05.

### Statistical Analysis

All continuous data following normal distribution were expressed as mean ± standard deviation (SD) and analyzed *via* Student’s t-test. All continuous data following skewed distribution were presented as median [interquartile range (IQR)] and analyzed *via* Mann-Whitney *U* test. Receiver operating characteristic (ROC) curves as well as the area under the ROC curves (AUC) were generated to determine the diagnostic value of miRNAs. Statistical analysis was performed using SPSS 19.0 (IBM, Armonk, NY) and GraphPad Prism 5 (Graphpad Software Inc., San Diego, CA). Differences were considered as statistically significant if *p* < 0.05.

## Results

### Study Patient Characteristics

The discovery cohort enrolled 3 sepsis patients at admission and three healthy controls for miRNA microarray analysis only. Later, thirty-seven patients with sepsis and 25 healthy controls were enrolled in the validation cohort. The demographic profile and clinical characteristics of the sepsis patients in the validation cohort were presented in Table 1. The most common etiologies for sepsis were pneumonia, peritonitis, and unknown causes. The 28-day hospital mortality rate for the enrolled sepsis patients was approximately 27.03% (10/37). Patients with sepsis were slightly older than healthy controls. However, there was no statistically significant difference in age between the two groups (*p* > 0.05).

**TABLE 1:** Demographic and clinical characteristics of sepsis patients in the validation cohort.

	Healthy controls *n* = 25	Sepsis n = 37	*p-*Value
Age (years)	64.00 ± 14.81	70.19 ± 19.76	>0.05
Male/female	12/13	23/14	
SOFA score		8.57 ± 3.32	
APACHE II score		22.70 ± 8.21	
Death, *n*		10	
Microbiology of patients (*n*)			
Gram-positive		2	
Gram-negative		11	
No organism cultured		24	
Type of infection (*n*)			
Pneumonia		14	
Severe cholangitis		1	
Peritonitis		7	
Other		15	
CRP, mg/L		100.39 ± 68.15	
PCT, mg/L		25.43 ± 29.48	

Data are presented as patient number or mean ± SD. APACHE II: acute physiology and chronic.

Evaluation II; SOFA: sequential organ failure assessment; CRP: C-reactive protein; PCT: procalcitonin.

### Characterization of Plasma EVs From Sepsis Patients

**FIGURE 1:**
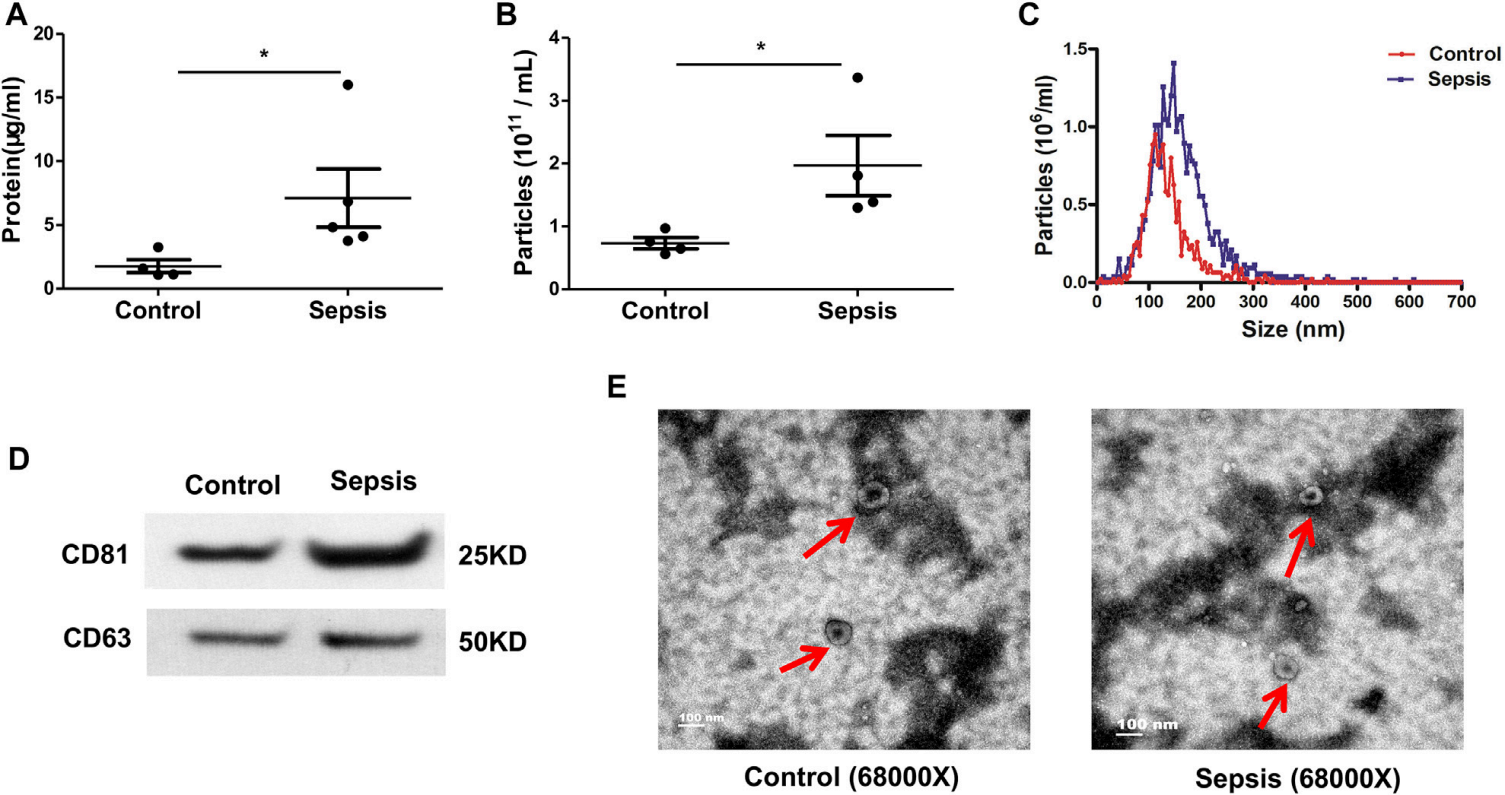
Characteristics of plasma-derived extracellular vesicles (EVs). Plasma-derived EVs were isolated from the plasma of sepsis patients and healthy controls *via* differential ultra-centrifugation. **(A)** BCA Protein Assay Kit was used to quantify the total protein levels of the EVs isolated from plasma. **(B, C)** Particle number and size distribution of EVs were analyzed *via* nanoparticle tracking analysis. **(D)** Plasma-derived EVs were assayed for expression of CD63 (band size of 25–60 kDa in literature depending on glycosylation) and CD81 (25 kDa) *via* Western blot. **(E)** Morphology of plasma-derived EVs was imaged by transmission electron microscopy. Data are presented as mean ± SD. *n* = 4–5. *p < 0.05.

Plasma samples were collected from sepsis patients and age-matched healthy controls. Plasma EVs were harvested by sequential centrifugation. The resulting pellets were resuspended in PBS and stored at −80°C. BCA Protein Assay Kit was used to determine the total protein concentration of EVs. EV particle number and size distribution were determined *via* nanoparticle tracking analysis. Plasma EVs from sepsis patients had higher protein contents (*p* < 0.05) (Figure 1A) and vesicle numbers (*p* < 0.05) (Figure 1B) compared with healthy controls. The size distribution showed a slightly larger size on average in the sepsis group with a peak at approximately 145 nm in diameter and range between 50 and 300 nm (Figure 1C). Both plasma EVs expressed traditional EV markers CD63 and CD81 (Figure 1D). TEM analysis presented a cup-shaped morphology of isolated plasma EVs (Figure 1E).

### EVs From Sepsis Patients Have Increased Expression of miR-483-3p, Let-7d-3p, and miR-92b-5p

**FIGURE 2:**
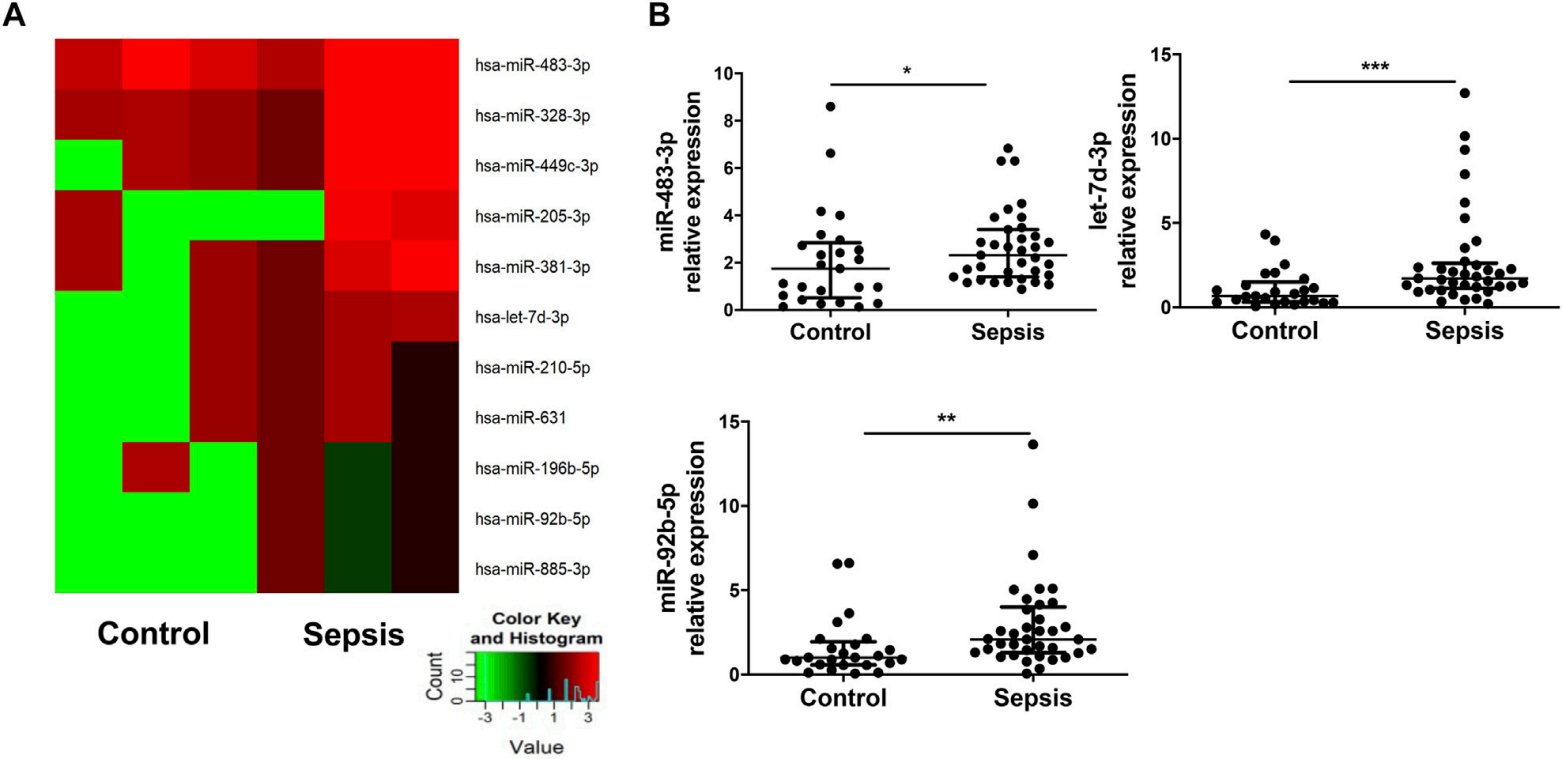
Elevated levels of miR-483-3p, let-7d-3p, and miR-92b-5p in EVs from sepsis patients. **(A)** miRNA profile of plasma EVs from sepsis patients and heathy controls from the discovery cohort was examined *via* microRNA array and compared between the two groups (*n* = 3). Eleven miRNAs demonstrated greater than 2-fold increase (*p* value between 0.001 and 0.12) in sepsis patients were chosen for hierarchical cluster analysis to produce the heat map. Green and red colors on the heat map denote a decrease and increase in miRNA expression, respectively. The color intensities correspond to relative expression levels. **(B)** The 6 of 11 miRNAs relating inflammation and injury were analyzed using qRT-PCR in a larger set of EV samples from the validation cohort. Three of the 6 miRNAs had elevated levels in sepsis group. The plot showed a median, interquartile range, and the individual data points. *n* = 25 for control. *n* = 35–37 for sepsis. *p < 0.05, **p < 0.01, ***p < 0.001.

A preliminary miRNA microarray of plasma EVs from 3 sepsis patients at admission and three healthy controls from the discovery cohort detected a total of 2,549 miRNAs. The miRNA sequencing data have been deposited in the NCBI Gene Expression Omnibus under accession number GSE184803. Among the detected miRNAs, 11 miRNAs were identified with greater than 2-fold upregulation (Figure 2A), whereas 29 miRNAs were downregulated > 2-fold (Supplemental Table S1). We chose to focus on the upregulated miRNAs. It has been reported that 6 out of the 11 upregulated miRNAs, including miR-483-3p, miR-328-3p, let-7d-3p, miR-92b-5p, miR-381-3p, and miR-210-5p, are involved in injury and inflammation (Lin et al., 2019; Fu et al., 2020; Maemura et al., 2020; Resaz et al., 2020). Subsequently, plasma EVs from the validation cohort of 37 sepsis patients at admission and 25 healthy controls were examined for the expression of the 6 inflammation-related miRNAs *via* qRT-PCR. Elevated expression of miR-483-3p (*p* < 0.05), let-7d-3p (*p* < 0.001), and miR-92b-5p (*p* < 0.01) (Figure 2B) was revealed in EVs from sepsis patients but not miR-328-3p, miR-381-3p, and miR-210-5p (data not shown).

### Polymicrobial Sepsis Enhances the Levels of miR-483-3p, Let-7d-3p, and miR-92b-5p in Plasma EVs

**FIGURE 3:**
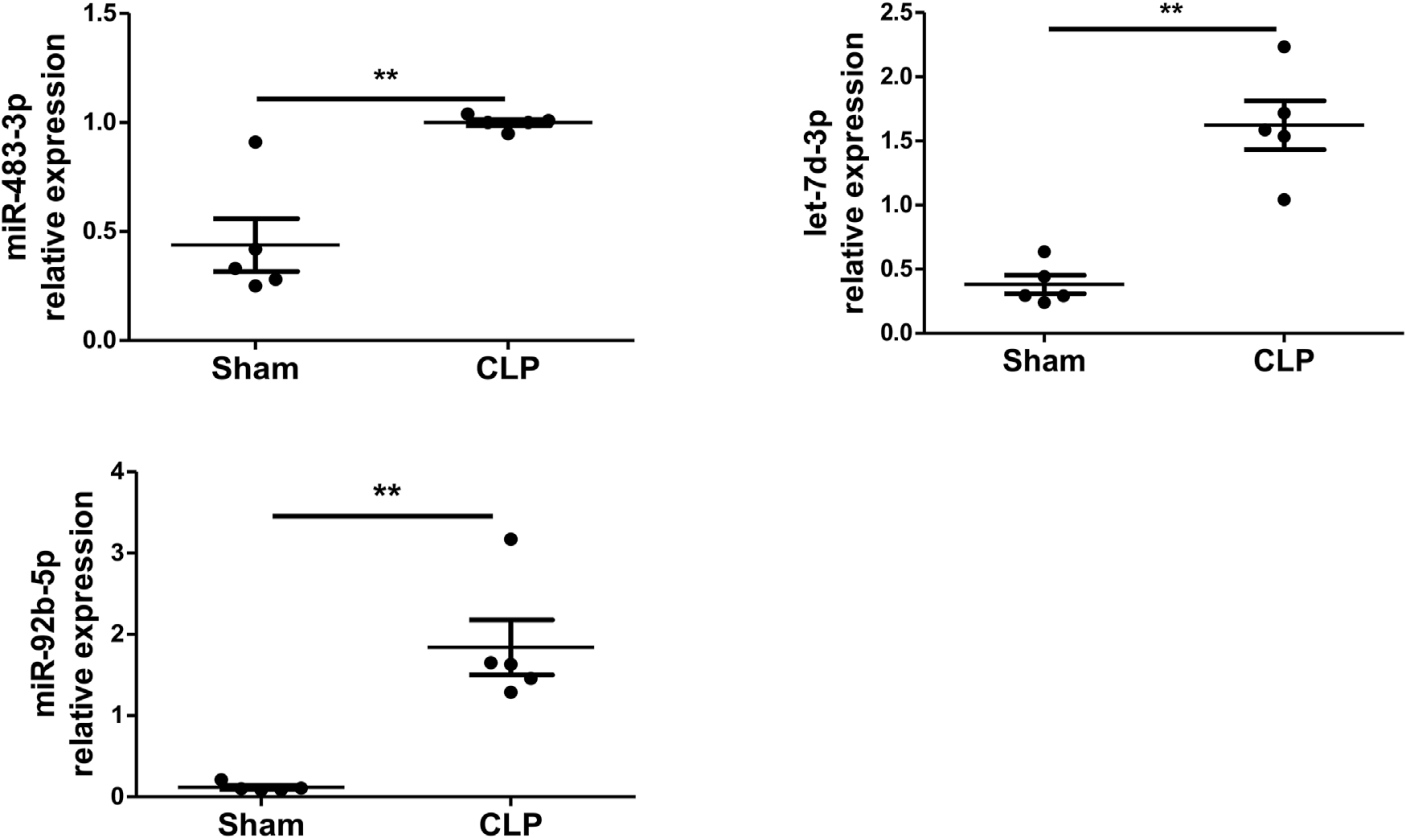
miR-483-3p, let-7d-3p, and miR-92b-5p expression in plasma EVs using a mouse model of sepsis. Plasma EVs were isolated from C57BL/6 mice after 24 h of sham or CLP surgery. Expression of miR-483-3p, let-7d-3p, and miR-92b-5p was determined *via* qRT-PCR. Data are presented as mean ± SD, *n* = 5. **p < 0.01.

To examine whether polymicrobial sepsis alters the expression of miR-483-3p, let-7d-3p, and miR-92b-5p, a CLP-induced mouse model of sepsis was introduced into the study. Plasma EVs were harvested at 24 h after CLP and examined for miR-483-3p, let-7d-3p, and miR-92b-5p levels *via* qRT-PCR. As shown in Figure 3, polymicrobial sepsis significantly increased the expression of miR-483-3p (*p* < 0.01), let-7d-3p (*p* < 0.01), and miR-92b-5p (*p* < 0.01) at 24 h after CLP surgery compared with sham operation. These data demonstrate that miR-483-3p, let-7d-3p, and miR-92b-5p levels are induced by polymicrobial sepsis and may serve as early biomarkers for sepsis.

### Let-7d-3p and miR-483-3p Levels in Plasma EVs From Sepsis Patients Strongly Associate With Disease Severity Scores and Existing Sepsis Biomarkers

**FIGURE 4:**
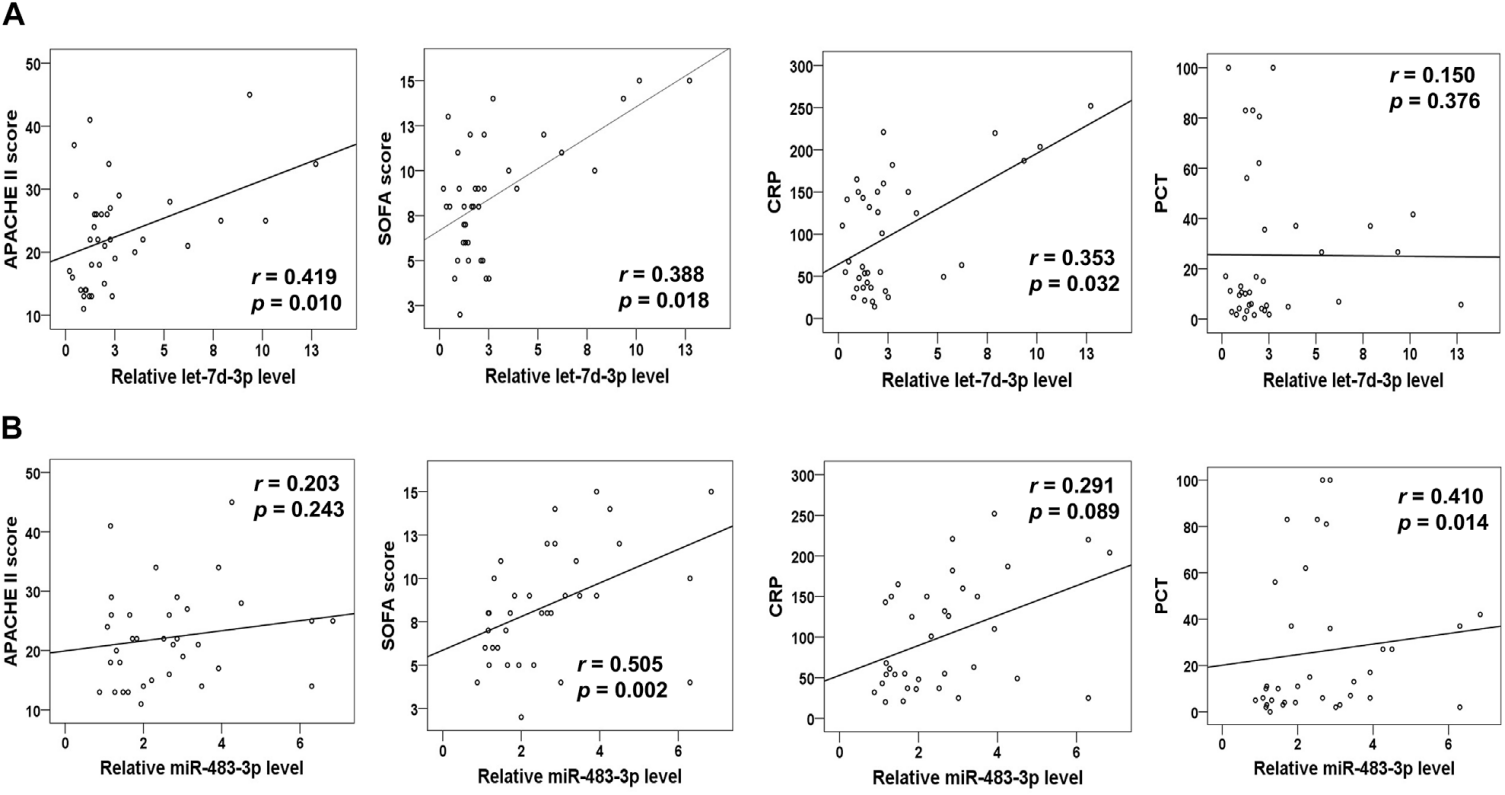
Correlation analysis of plasma EV miRNA levels (miR-483-3p and let-7d-3p) with disease severity scores and biomarkers for sepsis. **(A)** The curve was plotted by let-7d-3p relative expression values at admission of sepsis patients to their individual APACHE II and SOFA scores as well as CRP and PCT values. **(B)** The curve was plotted by miR-483-3p relative expression values at admission of sepsis patients to their individual APACHE II and SOFA scores as well as CRP and PCT levels. Each circle represents an individual patient. *n* = 35–37.

Next, our goal was to elucidate the clinical relevance between the levels of miR-483-3p, let-7d-3p, and miR-92b-5p in plasma EVs and sepsis. Spearman’s correlation analysis was performed between levels of miR-483-3p, let-7d-3p, and miR-92b-5p and the critical care scoring systems including Acute Physiology and Chronic Evaluation II (APACHE II) as well as SOFA. Strong positive linear relationships existed between levels of let-7d-3p and APACHE II score (*r* = 0.419, *p* = 0.010) as well as SOFA score (*r *= 0.388, *p* = 0.018) (Figure 4A). Let-7d-3p expression significantly correlated to serum levels of CRP (*r* = 0.353, *p* = 0.032) but not PCT (*r* = 0.150, *p* = 0.376), two popular biomarkers of sepsis. In contrast, miR-483-3p expression displayed a significant correlation with SOFA score (*r* = 0.505, *p* = 0.002) and PCT level (*r* = 0.410, *p* = 0.014), but not APACHE II score (*r* = 0.203, *p* = 0.243) and CRP level (*r* = 0.291, *p* = 0.089) (Figure 4B). However, miR-92b-5p showed no correlation with any of the above disease severity scores or existing biomarkers (data not shown). Therefore, our study was focused on miR-483-3p and let-7d-3p.

### Combination of Plasma EV miR-483-3p and Let-7d-3p has Diagnostic Performance for Sepsis

**FIGURE 5:**
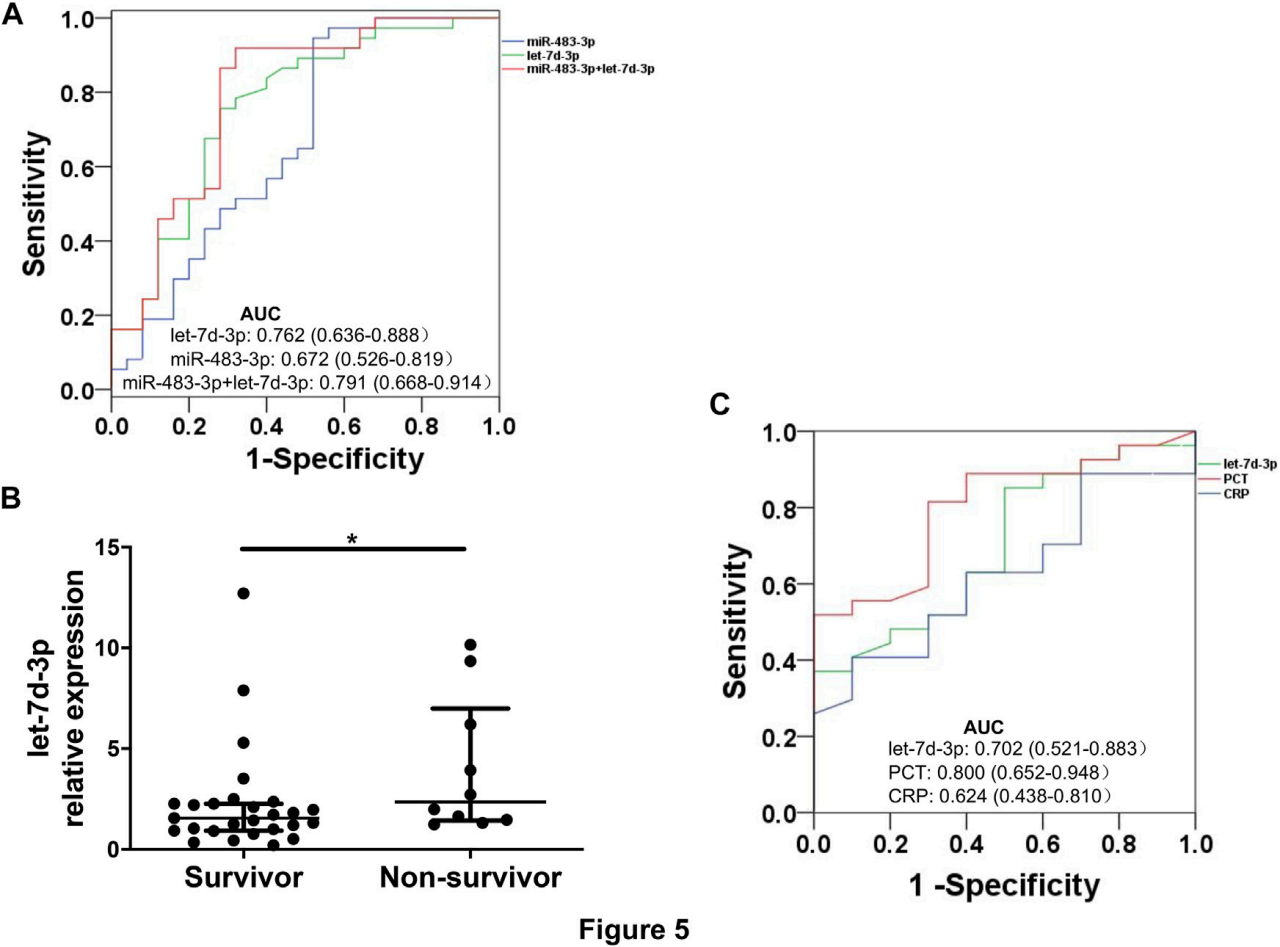
ROC curves for sepsis diagnosis and mortality prediction. **(A)** ROC curves show sensitivity and specificity of plasma EV miR-483-3p and let-7d-3p, alone or in combination, for sepsis diagnosis. **(B)** Levels of plasma EV let-7d-3p were compared between non-survivors (*n* = 10) and survivors (*n* = 27). The plot showed a median, interquartile range, and the individual data points. *p < 0.05. **(C)** ROC curves show sensitivity and specificity of plasma EV let-7d-3p, PCT, and CRP for predicting 28-day mortality.

The diagnostic value of miR-483-3p and let-7d-3p was examined by ROC curve analysis to discriminate between sepsis patients and heathy controls. Individually, let-7d-3p had an AUC of 0.762 (95% CI 0.636–0.888, *p* = 0.001), while the AUC for miR-483-3p was 0.672 (95% CI 0.526–0.819, *p* = 0.022) (Figure 5A). A combined ROC curve with miR-483-3p and let-7d-3p provided additional benefit to the diagnostic performance (AUC = 0.791, 95% CI 0.668–0.914, *p* = 0.001).

### Plasma EV Let-7d-3p has a Trend Towards Statistical Significance in Predicting 28-day Mortality

All enrolled patients were monitored for 28 days after enrollment or until death. Levels of plasma EV let-7d-3p, but not miR-483-3p, were significantly higher in non-survivors (*n* = 10) than in survivors (*n* = 27) (*p* < 0.05) (Figure 5B). The performance of EV let-7d-3p, PCT, and CRP for predicting 28-day mortality was evaluated by ROC curve analysis to discriminate between hospital deaths and survivors. PCT performed the best for predicting 28-day mortality (AUC = 0.800, 95% CI 0.652–0.948, *p* = 0.006) (Figure 5C). Satisfactory performance was observed in let-7d-3p (AUC = 0.702, 95% CI 0.521–0.883, *p* = 0.062) and CRP (AUC = 0.624, 95% CI 0.438–0.810, *p* = 0.252) but did not reach statistical significance due to the small sample size.

### miR-483-3p and Let-7d-3p Modulate Sepsis *via* Multiple Pathways

**FIGURE 6:**
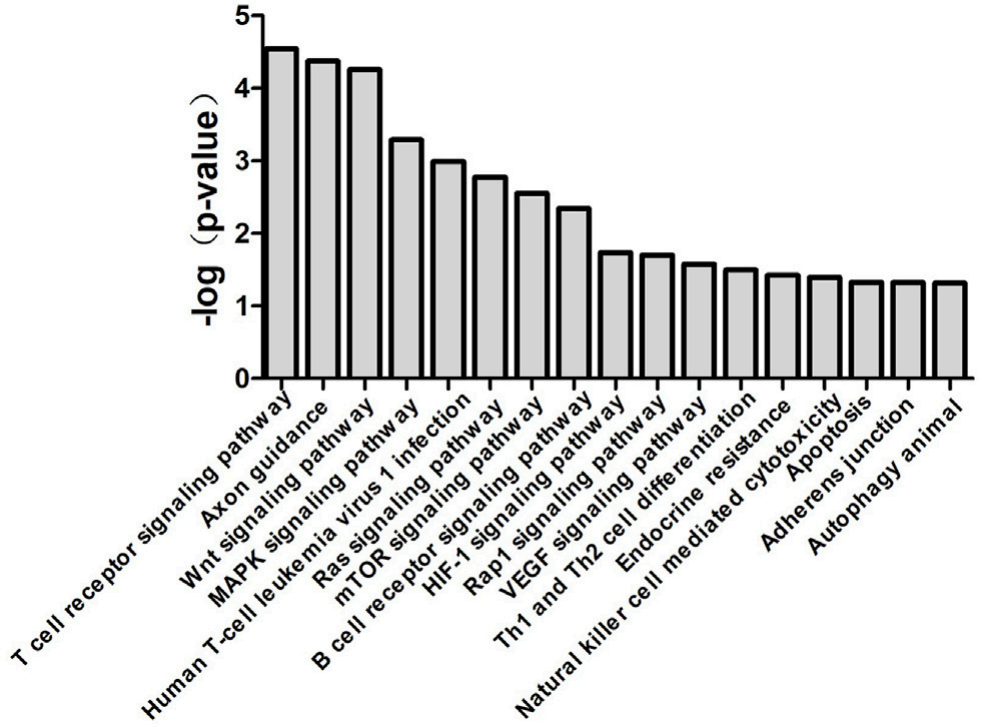
Canonical pathways associated with miR-483-3p and let-7d-3p generated by bioinformatics analysis. Top 17 over-represented biological pathways for gene targets of elevated miR-483-3p and let-7d-3p were depicted in the bar graph. Data are presented as the negative log *p*-value generated from the Fisher’s exact test. Pathways which were more closely associated with the gene targets had higher values.

miR-483-3p and let-7d-3p were input into miRDB and TargetScan databases to generate target genes of each miRNA. KEGG analysis was carried out by uploading the predicted target genes. Pathways with *p* value of <0.05 from Fisher’s exact test were recognized as significant (Figure 6). The associated pathways included innate immune response (cytotoxicity of natural killer cells, endotoxin-stimulated MAPK, mTOR, and HIF-1), adaptive immune response (T cell receptor signaling, B cell receptor signaling, and Th1/Th2 differentiation), endothelial dysfunction (VEGF pathway and apoptosis), and endocrine resistance to sepsis.

**FIGURE 7:**
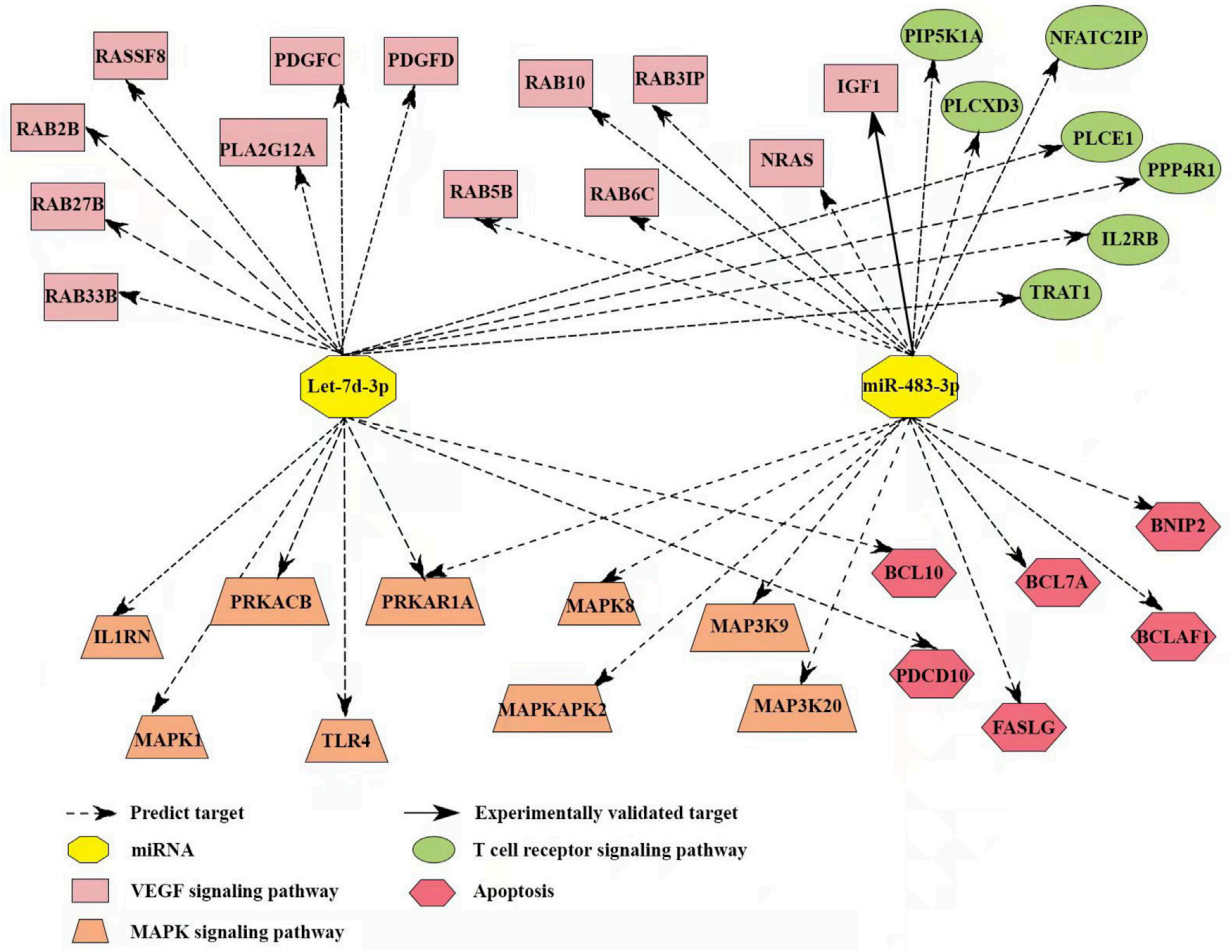
Target gene network of miR-483-3p and let-7d-3p in pathways with relevance to immune and endothelial dysfunctions during sepsis. The network contained 35 targeted genes and was constructed using reported and predicted human miRNA:mRNA interactions from the TargetScan and miRDB databases.

To understand the mechanism of miR-483-3p and let-7d-3p in the pathogenesis of sepsis, TargetScan and miRDB databases were reviewed to identify the target mRNAs. miR-483-3p and let-7d-3p are associated with 35 target genes in pathways highly relevant to sepsis (Figure 7). One of the target genes has been experimentally validated (solid line), whereas the others are predicted targets. Among the 35 targets, 9 genes are correlated with MAPK signaling pathway, which is pivotal for proinflammatory immune response. Thirteen other genes are associated with VEGF signaling pathway, which is essential for regulating endothelial barrier integrity. In addition, 6 target genes play a role in cell apoptosis while 7 target genes modulate T cell receptor signaling pathway, which contributes to T cell dysfunction in sepsis.

### Potential Target Genes of miR-483-3p and Let-7d-3p are Validated by qRT-PCR

**FIGURE 8:**
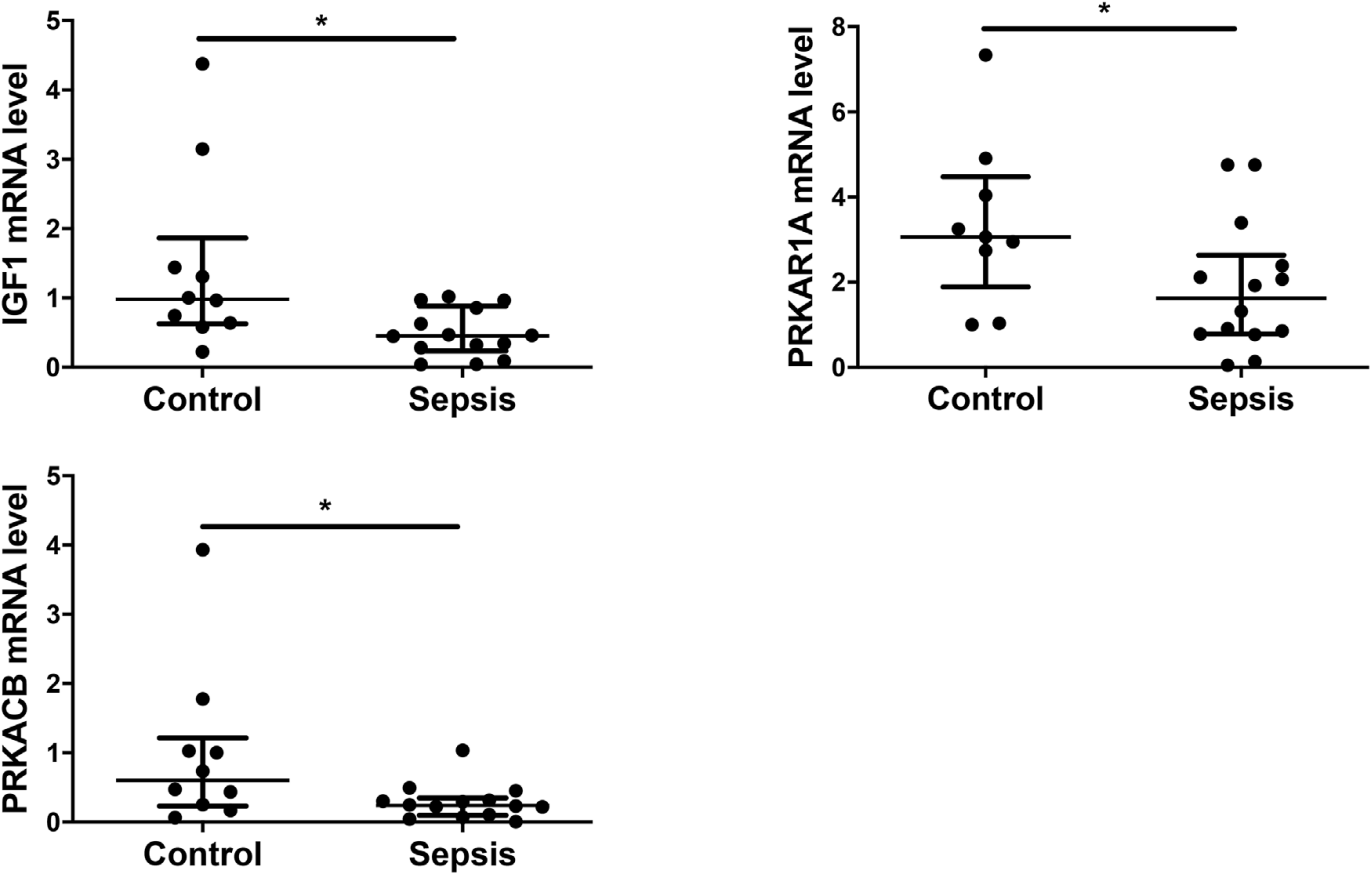
Experimental validation of selected target genes of miR-483-3p and let-7d-3p. Plasma EVs were analyzed for differentially expressed genes from a set of 8 targets *via* qRT-PCR. The values were relative mRNA abundance of IGF1, PRKAR1A, and PRKACB in EVs. β-actin mRNA was used as an internal control. The plot showed a median, interquartile range, and the individual data points. *n* = 9–10 for control. *n* = 14 for sepsis. *p < 0.05.

Eight genes, including PDGFC, PDGFD, IGF1, IL2RB, PRKAR1A, PRKACB, BCL10, and BNIP2, were selected from the 4 sepsis-associated pathways listed in Figure 7 and examined for their transcript levels in the plasma EVs. qRT-PCR analysis showed that levels of IGF1, which promotes angiogenesis *via* VEGF signaling pathway, were downregulated in sepsis patients (Figure 8). In the meantime, the expression of PRKAR1A and PRKACB, which are negative regulators for MAPK pathway, was also decreased in the sepsis cohort, while the expression of the other 5 genes was not significantly altered. These results reveal that IGF1, PRKAR1A, and PRKACB are possible targets of miR-483-3p and let-7d-3p in sepsis.

## Discussion

To our knowledge, the present study is the first to report that plasma EV miRNAs have the potential to serve as biomarkers for the diagnosis of sepsis. Our results showed that plasma EVs from sepsis patients presented higher protein contents and vesicle numbers than those from healthy controls. These EVs had increased expression of miR-483-3p and let-7d-3p compared with EVs of healthy controls. Elevated expression of these two miRNAs was also demonstrated in a mouse model of polymicrobial sepsis. There was a significant positive correlation between the two miRNA levels and disease severity scores, as well as existing biomarkers for sepsis. In addition, the combination of miR-483-3p and let-7d-3p may serve as a diagnostic biomarker for sepsis. Furthermore, pathway analysis revealed that the two miRNAs may regulate sepsis *via* multiple pathways.

There are a small number of studies examining miRNAs in EVs relating to sepsis. Plasma EVs from patients with septic shock conveyed miRNAs regulating inflammatory response and cell cycle (Real et al., 2018). Another group found that circulating plasma EVs from septic mice mediated inflammation *via* miRNA-dependent mechanisms (Xu et al., 2018). Additionally, plasma EVs from septic mice significantly decreased deformability of red blood cells and showed distinct miRNA profiles (Subramani et al., 2018). In EVs separated from the supernatant of platelets treated with saline or LPS, levels of miR-24-3p, miR-15b-5p, miR-25-3p, miR-126-3p, miR-378a-3p, and miR-155-5p were significantly higher in LPS group than in saline group (Jiao et al., 2020). In another study with mouse model of sepsis, EVs from intestinal luminal lavage were enriched with miRNAs, which putatively targeted TNF-α and IL-17A (Appiah et al., 2020).

Many studies have compared the diagnostic potential of miRNAs in peripheral blood and circulating EVs. In patients with myelodysplastic syndromes, miRNAs in plasma EVs provided better biomarkers for patient survival, while miRNAs in plasma were predictive of the response to azacitidine treatment (Hrustincova et al., 2020). In blood samples from healthy controls, EVs were enriched with miRNAs and had more consistent expression profile than plasma and serum (Cheng et al., 2014). Another group found that plasma miR-375 could distinguish between prostate cancer and benign prostatic hyperplasia, whereas miR-200c-3p and miR-21-5p in EVs had better diagnostic performance (Endzelins et al., 2017). In two animal models of kidney diseases, expression profiles of miRNAs in plasma and EVs were dramatically different (Xie et al., 2017). Another study compared miRNAs in plasma and corresponding EVs from 12 children with retinoblastoma and 12 healthy controls. They detected an average of 537 miRNAs in plasma and 625 in EVs, as well as a plasma signature of 19 miRNAs to discriminate patients from controls (Castro-Magdonel et al., 2020). These results suggest that miRNAs in peripheral blood and circulating EVs do not mirror one another, instead, they represent unique facets of the disease.

Current literature provides evidence that miR-483-3p and let-7d-3p regulate gene expression *via* multiple signaling pathways. One study demonstrated that wound closure was preceded by an elevation of miR-483-3p, which triggered cell cycle arrest in early G1 phase (Bertero et al., 2013). Another group showed that EV miR-483-3p from bronchoalveolar lavage mediated innate immune response during influenza virus infection (Maemura et al., 2018). The group also documented that circulating EV miR-483-3p levels were significantly increased when mice were infected with avian H5N1 influenza virus. They hypothesized that miR-483-3p-containing EVs were transferred to vascular endothelial cells and induced the upregulation of inflammatory cytokines (Maemura et al., 2020). Another study documented that elevated expression of miR-483-3p inhibited the endothelial response to injury in Type 2 diabetes in mice (Kuschnerus et al., 2019). In contrast, circulating let-7d-3p was shown to be significantly upregulated in pigs infected with the porcine whipworm (Hansen et al., 2016). Circulating endothelial EVs from cigarette-smoke exposed mice were enriched in let-7d and reduced the removal of apoptotic cells by phagocytotic macrophages (Serban et al., 2016). There was an inverse correlation between let-7d level and microvessel density in tissues of renal cell carcinoma. Moreover, let-7d suppressed intratumoral macrophage M2 polarization and targeted IL-10 (Su et al., 2020).

In the present study, our results demonstrated that miR-483-3p and let-7d-3p were upregulated in early sepsis. The mechanism of such regulation remains unclear. In monocytes, Rossato et al. (2012) reported that LPS induced miR-187 expression, which negatively modulated TNF-α, IL-6, and IL-12p40 production, *via* an IL-10–dependent manner. In another study of LPS-stimulated monocytes, miR-146b expression was elevated *via* an IL-10-mediated STAT3-dependent loop. In addition, miR-146b modulated the TLR4 signaling pathway *via* targeting TLR4 and several key signaling proteins (Curtale et al., 2013). Another group discovered that LPS-induced microRNA-146 promoted a feed-forward loop that interfered TNF-α and IL-6 synthesis in THP-1 monocytes (Brudecki et al., 2013). Tili et al. revealed that levels of miR-125b and miR-155 oscillated rapidly in Raw 264.7 macrophages following LPS and TNF-α stimulation. In addition, the NF-ĸB activity was required for the phenomenon (Tili et al., 2007). The molecular mechanism underlying altered expression of miR-483-3p and let-7d-3p in sepsis warrants further investigation.

Our study had several limitations worth noting. First, the results should be regarded as preliminary and hypothesis-driven, given the limited sample size of the present study. The small sample size may help to explain why miR-483-3p was not significantly higher in non-survivors comparted to survivors. Secondly, an uninfected control group of ICU patients was not included into the study. Therefore, this study could not determine whether the plasma EV miRNAs could discriminate sepsis from noninfectious insult. Thirdly, this investigation predicted multiple targets and pathways that might mediate the effects of miR-483-3p and let-7d-3p. However, function analysis was not performed to test the proposed targets and pathways.

In summary, the present study identified that miR-483-3p and let-7d-3p were elevated in the plasma EVs of sepsis patients compared to healthy controls. A combination of plasma EV miR-483-3p and let-7d-3p may discriminate sepsis from healthy state. However, a larger multicenter study is warranted to establish the value of such plasma EV-based miRNA biomarkers for sepsis diagnosis and prognosis. In addition, studies examining the role of plasma EV miR-483-3p and let-7d-3p in the pathogenesis of sepsis have the potential to generate crucial knowledge for sepsis diagnosis and treatment.

## Data Availability

The datasets presented in this study can be found in online repositories. The names of the repository/repositories and accession number(s) can be found in the article/Supplementary Material.
