# Supplementary material for: Diagnostic Potential of Plasma Extracellular Vesicle miR-483-3p and Let-7d-3p for Sepsis
Source: Front Mol Biosci. 2022 Feb 2;9:814240. doi: 10.3389/fmolb.2022.814240 (PMC8847446; doi:10.3389/fmolb.2022.814240)
Supplement: Supplementary file 1 [file Table1.DOCX]

**Online Data Supplement for**

**Diagnostic potential of plasma extracellular vesicle miR-483-3p and let-7d-3p for sepsis**

Guanguan Qiu**^1^**^†^, Jiajie Fan**^2^**^†^, Guoping Zheng**^1^**, Jiangping He**^1^**, Fangping Lin**^1^**, Menghua Ge**^1^**, Lanfang Huang**^1^**, Jiangmei Wang**^2^**, Jie Xia^2^, Ruoqiong Huang**^2^**, Qiang Shu**^2^**, Jianguo Xu**^2,1^**

**^1^** Shaoxing Second Hospital, 123 Yanan Road, Shaoxing, Zhejiang 312000

**^2^** Department of Thoracic and Cardiovascular Surgery, Children’s Hospital of Zhejiang University School of Medicine and National Clinical Research Center for Child Health, 3333 Binsheng Road, Hangzhou, Zhejiang 310052

**Supplemental Table 1. List of miRNAs with > 2-fold downregulation in plasma EVs from sepsis patients compared with healthy controls (p < 0.10).**

**Downregulated miRNAs**

| hsa-let-7d-5p  hsa-miR-106b-5p  hsa-miR-107  hsa-miR-141-3p  hsa-miR-181d-3p  hsa-miR-200b-5p  hsa-miR-25-5p  hsa-miR-298  hsa-miR-302d-5p  hsa-miR-331-3p  hsa-miR-378a-3p  hsa-miR-422a  hsa-miR-490-3p  hsa-miR-513b-3p  hsa-miR-513b-5p  hsa-miR-513c-5p  hsa-miR-514a-5p  hsa-miR-520b  hsa-miR-520e  hsa-miR-525-5p  hsa-miR-563  hsa-miR-584-5p  hsa-miR-608  hsa-miR-622  hsa-miR-654-5p  hsa-miR-767-3p  hsa-miR-877-5p  hsa-miR-943  hsa-miR-98-3p |
| --- |
